# Immune Responses to Pandemic H1N1 Influenza Virus Infection in Pigs Vaccinated with a Conserved Hemagglutinin HA1 Peptide Adjuvanted with CAF^®^01 or CDA/αGalCerMPEG

**DOI:** 10.3390/vaccines9070751

**Published:** 2021-07-06

**Authors:** Sergi López-Serrano, Lorena Cordoba, Mónica Pérez-Maillo, Patricia Pleguezuelos, Edmond J. Remarque, Thomas Ebensen, Carlos A. Guzmán, Dennis Christensen, Joaquim Segalés, Ayub Darji

**Affiliations:** 1 IRTA, Centre de Recerca en Sanitat Animal (CReSA), Campus de la Universitat Autònoma de Barcelona, 08193 Bellaterra, Spain; sergi.lopez@irta.cat (S.L.-S.); lorena.cordoba@irta.cat (L.C.); monica.perez@irta.cat (M.P.-M.); patricia.pleguezuelos@irta.cat (P.P.); joaquim.segales@irta.cat (J.S.); 2 Biomedical Primate Research Center, Department of Immunobiology, P.O. Box 3306, 2280 GH Rijswijk, The Netherlands; remarque@bprc.nl; 3 Helmholtz Centre for Infection Research, Department of Vaccinology and Applied Microbiology, Inhoffenstraße 7, 38124 Braunschweig, Germany; thomas.ebensen@helmholtz-hzi.de (T.E.); Carlos.Guzman@helmholtz-hzi.de (C.A.G.); 4 Virus Research and Development Laboratory, Department of Virus and Microbiological Special Diagnostics, Statens Serum Institute, Artillerivej 5, 2300 Copenhagen, Denmark; den@ssi.dk; 5 OIE Collaborating Centre for the Research and Control of Emerging and Re-Emerging Swine Diseases in Europe (IRTA-CReSA), 08193 Bellaterra, Spain; 6 Departament de Sanitat i Anatomia Animals, Facultat de Veterinària, UAB, 08193 Bellaterra, Spain

**Keywords:** vaccine, adjuvants, influenza, immunity, pathology

## Abstract

This study aimed to evaluate the immune response and protection correlates against influenza virus (IV) infection in pigs vaccinated with the novel NG34 HA1 vaccine candidate adjuvanted with either CAF^®^01 or CDA/αGalCerMPEG (αGCM). Two groups of six pigs each were vaccinated intramuscularly twice with either NG34 + CAF^®^01 or NG34 + CDA/αGCM. As controls, groups of animals (*n* = 6 or 4) either non-vaccinated or vaccinated with human seasonal trivalent influenza vaccine or NG34 + Freund’s adjuvant were included in the study. All animal groups were challenged with the 2009 pandemic (pdm09) strain of H1N1 (total amount of 7 × 10^6^ TCID_50_/mL) via intranasal and endotracheal routes 21 days after second vaccination. Reduced consolidated lung lesions were observed both on days three and seven post-challenge in the animals vaccinated with NG34 + CAF^®^01, whereas higher variability with relatively more severe lesions in pigs of the NG34 + CDA/αGCM group on day three post-infection. Among groups, animals vaccinated with NG34 + CDA/αGCM showed higher viral loads in the lung at seven days post infection whereas animals from NG34 + CAF^®^01 completely abolished virus from the lower respiratory tract. Similarly, higher IFNγ secretion and stronger IgG responses against the NG34 peptide in sera was observed in animals from the NG34 + CAF^®^01 group as compared to the NG34 + CDA/αGCM. NG34-vaccinated pigs with adjuvanted CAF^®^01 or CDA/αGCM combinations resulted in different immune responses as well as outcomes in pathology and viral shedding.

## 1. Introduction

Influenza is a contagious disease caused by Influenza viruses (IV) that mainly can affect birds, which represent the natural reservoir and mammals that act as natural hosts [1]. Within mammals, IV can cause disease in a wide range of groups including carnivores, human and pigs [2]. Although only three subtypes of Influenza A viruses (IAV) make up the vast majority of influenza infections in pigs worldwide (H1N1, H1N2 and H3N2) [3], the high variability between strains makes the production of efficacious vaccines for the prevention and control of the disease a big challenge to vaccine-manufacturers. Swine influenza viruses (SwIV) not only cause significant economic losses for the swine industry, but also are important zoonotic pathogens since variant viruses in swine pose threat for humans, e.g., H1N1 2009 pandemics. Moreover, swine represent a model of choice for the research of Influenza infection and immunity among other animals like mice and ferrets [4].

Vaccination is considered the most important and effective strategy to prevent and control IAV infection and disease in both animals and humans. Current strategies to combat IAV infection include vaccines that consist seasonal trivalent/quadrivalent Influenza Virus (IV) strains, based on inactivated virus or its corresponding hemagglutinins with or without additional adjuvants [5]. The immune responses triggered by these vaccines; however, these are strain specific and do not protect individuals against heterologous emerging strains, because of the characteristic mutating nature of IVs. Multivalent or universal vaccines, based on conserved antigen motifs from influenza virus, could be an attractive albeit challenging strategy to broadly prevent influenza virus infection and reduce the risk of influenza pandemics [6,7,8]. Conserved antigen subunits, on the other hand, are often poor immunogens and may require additional adjuvants to induce the strong humoral and cellular immune responses needed to overcome IV infection [9]. 

Our research group has extensively worked to identify and select biologically active antigen subunits from Hemagglutinin 1 (HA1) of IAV for the development of a universal vaccine. Using an informational spectrum method (ISM) [10], a 34 amino acid antigen subunit (NG34) from HA1 of IAV was selected as a potential vaccine candidate. NG34 is located within the immunogenic site E in the N terminus of HA1, a domain close to receptor binding site of the HA characterized as well conserved. Recently, we have demonstrated that immunization with the NG34 antigen either incorporated in a plasmid or as a peptide formulated with adjuvants like Montanide, Diluvac Forte, Addavax or Alhydrogel induced specific antibodies as well as CD4 T cell responses, that conferred protection against homologous and heterologous IV infection in a pig model. The robust characteristic of this immunogenic NG34 peptide has been demonstrated in different experimental animal infection models [11,12]. 

In the present study we examined the immune correlates that may define protection against IV infection in pigs immunized with the NG34 peptide adjuvanted with either a liposome based “Cationic Adjuvant Formulation 01” (CAF^®^01) or a combination of bis-(3,5)-cyclic dimeric adenosine monophosphate (CDA) and α-galactosylceramide methoxypolyethylene glycol (αGCM). The adjuvant CAF^®^01 is composed of ammonium surfactant N,N’-dimethyl-N,N’-dioctadecylammonium (DDA) and C-type lectin receptor (Mincle) agonist α,α’-trehalose 6,6’-dibehenate (TDB) a syntethic glycolipid analog to the cord factor from *Mycobacterium tuberculosis*. CAF^®^01 is known to induce Th1/Th17 type cell mediated immunity as well as strong humoral responses [13]. It has furthermore been shown to effectively improve TIV efficacy both against homologous and heterologous IAV infection [14,15]. CDA is a monocyclic dinucleotide secreted by *Listeria monocytogenes* that is known to activate the “Stimulator of Interferon Genes” (STING) in the host, leading to the activation of TNF and type I IFN that stimulate Th1/Th2/Th17 and cytotoxic cellular and humoral immune responses [16]. The αGCM is a pegylated glycolipid derived from the marine sponge *Agelas mauritianus* and is a superagonist for iNKT cells involved in immune-modulation, stimulation of the Th2 response and enhancement of mucosal antibody response [17]. In addition to the mentioned adjuvants, two groups of pigs were treated, respectively, with Seasonal Trivalent Influenza Vaccine (TIV) and NG34 + Freund’s Adjuvants used as historical controls to evaluate their effectivity and immunogenicity.

## 2. Materials and Methods

### 2.1. Vaccine Antigens and Adjuvants

NG34 peptide, which sequence corresponds with 34 amino acids located in the E site from the N terminus of HA1 from A/Catalonia/63/2009 strain (pdm09 H1N1, positions 87 to 120 [GenBank ACS36215], was synthesized by CASLO ApS (Kongens Lyngby, Denmark). The lyophilized NG34 was reconstituted to a concentration of 2 mg/mL in ammonium chloride solution following manufacturer’s instructions and stored at −80 °C until use. Integrity of the peptide sequence was confirmed before the experiment by MALDI-TOF mass spectrometry. 

CAF^®^01 and CDA/αGCM adjuvants were provided by the collaborator laboratories of this study, the Statens Serum Institut (Copenhagen, Denmark) and Helmholtz-Zentrum für Infektionsforschung (Braunschweig, Germany), respectively. Vaccine formulations were prepared according to its instructions in the recommended proportions (Table 1). Complete (CFA) and incomplete (IFA) Freund’s Adjuvant were purchased from Sigma Aldrich (Madrid, Spain). Animals primed with CFA and boosted with IFA adjuvant in combination with NG34 were considered as a positive control group for the adjuvants. 

Unadjuvanted human TIV vaccine used in the Portugal and Spain influenza vaccination campaign of 2018–2019 (*Chiroflu^®^*, Seqirus Srl., Siena, Italy) was used in this study to benchmark against an approved vaccine. Its composition comprised 15 µg of the three hemagglutinins from the following strains produced in egg: A/Singapore/GP1908/2015 (similar strain to A/Michigan/45/2015 (H1N1) pdm09); A/Singapore/INFIMH-16-0019/2016 (H3N2) and B/Maryland/15/2016 wild type (similar strain to B/Colorado/06/2017). 

### 2.2. Cell Cultures and Virus

Madin-Darby Canine Kidney cells (MDCK, ATCC CCL-34) were used for virus propagation, titration and seroneutralization assays; cells were cultured in Dulbecco’s Modified Eagle Medium DMEM (Lonza, Basel, Switzerland) supplemented with 5% of fetal bovine serum (Euroclone, Milan, Italy), 1% of L-glutamine (Gibco Life Technologies, Madrid, Spain) and 1% penicillin-streptomycin (Gibco Life Technologies, Madrid, Spain) at 37 °C 5% CO_2_.

A/Human/Catalonia/063/2009 pandemic (pdm09) H1N1 Influenza virus available in the laboratory was propagated in MDCK cells. Briefly, monolayer cell cultures were inoculated with the help of 10 µg/mL porcine trypsin (Sigma-Aldrich, Madrid, Spain) at a MOI of 0.0001 to obtain the desired concentration at the harvest two days later. Subsequently, cultures were frozen to rupture infected cells and centrifuged. Supernatants were stored until use at −80 °C. Titration of inocula were performed by culture of serial dilutions in MDCK cells and the resulting TCID_50_/mL was calculated using the Reed and Muench method [18]. 

Some volume of viral production was UV light inactivated under a lamp to use for cell stimulation. Briefly, 1 mL volume of viral stock was dispensed in a six-well cell culture plate to reach a 1 mm of thickness under the UV light for 20 min. This procedure was repeated until the desired volume was inactivated. Once obtained, viral bulk was tested for viability by serial dilution cultures and read by cytopathic effect as it is described above. Inactivated viral volumes were aliquoted and frozen at −80 °C until use. 

### 2.3. Experimental Design

Clinically healthy Landrace x Large White pigs from livestock farms (Selecció Batallé, Girona, Spain) of about six weeks-of-age and similar weight (10–13 kg) were firstly screened for Influenza NP protein antibodies by ID Screen^®^ Influenza A Antibody Competition ELISA (IDVET, Grabels-Montpellier, France). Thirty-four seronegative animals were selected and then distributed randomly into six experimental groups of six or four pigs blocking by obtained ELISA titer (Table 1). All groups were split in two boxes at BSL3 facilities of IRTA-CReSA with three animals per group in each box; therefore, all groups were represented within the same box, sharing the air space. Prior to the experiment all animals were confirmed negative for IV twice (at the selection and before the beginning of the experiment) using RT-qPCR [19] (see below, RT-qPCR–Viral load section) to ensure they were not exposed to IVs. 

Animals were immunized on study days 0 and 21; 39 days after the first vaccination, animals were challenged with pdm09 H1N1 IV strain by two routes: intranasally using a nebulizer with 1 mL of 10^6^ TCID_50_/mL per nostril and by endotracheal route inoculating 5 mL of 10^6^ TCID_50_/mL. Uninfected animals were inoculated in both ways with sole viral propagation medium (DMEM 1% Gln 1% penicillin-streptomycin). Half of the animals per group were sacrificed on three days post-inoculation (dpi) and the remaining animals on day seven post-inoculation (p.i.). Euthanasia of the animals was performed by an intravenous overdose of sodium pentobarbital (140 mg/kg). During all experimental procedures, animals were fed ad libitum and were not treated with antibiotics, anesthetics, or analgesics since they were not suffering from any clinical condition that required such intervention.

### 2.4. Sampling 

Samples were collected at vaccination, challenge and necropsy, comprising two nasal swabs, clotted blood for sera and EDTA-treated whole blood for PBMCs. Blood was collected from the jugular vein with Vacutainer tubes (Becton Dickinson, CA, USA); sera were obtained by centrifuging the tubes 10 min at 2500 rpm (1258 g) at room temperature. Two nasal swabs collected from both nostrils were resuspended in 1 mL of PBS, supplemented with 1% penicillin-streptomycin (Gibco Fisher Scientific, Waltham, MA, USA). Post-inoculation, nasal swabs were collected daily until the end of the experiment. In addition, necropsy samples comprised portions of lung (apical, medial and cranial part of diaphragmatic left lobes), trachea and nasal turbinates for histopathological assessment conserved in 10% formalin. Bronchoalveolar lavage (BALF) was obtained dissecting the right lung and filling it with 150 mL of sterile PBS, recovering after a smooth massage a final volume of about 50 mL of lavage. Serum samples, nasal swabs and BALF were stored at −80 °C until use. 

### 2.5. Clinical Signs and Pathological Assessment

Rectal temperatures and flu-like clinical signs were evaluated throughout the whole experimental period. Fever was considered when rectal temperature values were above 40 °C. To assess gross lesions caused by infection, individual lungs were collected on necropsy days and pictures of dorsal and ventral sides were taken. The macroscopic affected area was quantified by image analysis (ImageJ online free software), a scoring system was applied as previously described [20]. 

Formalin fixed tissues were embedded in paraffin wax and sectioned 3–5 µm to stain with hematoxylin-eosin for histopathologic assessment and with monoclonal antibody from hybridoma ATCC No. HB-65 against AIV nucleoprotein for immunohistochemistry as it is described before [21]. 

### 2.6. Quantitative PCR RT-qPCR–Viral Load

Nasal shedding and viral load were assessed through quantitative RT-PCR for M protein as it has been previously described [19]. Viral RNA was extracted from resuspended nasal swabs and BALF using IndiMag Pathogen Kit (Indical Bioscience, Leipzig, Germany) following the manufacturer’s instructions. Subsequently, the TaqMan RT-qPCR mentioned before was run in Fast7500 Thermocycler equipment (Applied Biosystems, Foster City, CA, USA). 

Samples with undetectable fluorescence were considered negative. Genome equivalent copies were calculated per sample using a standard curve. An arbitrary Ct value of 39.5 (below the detection level of the technique) was given to those negative samples for statistical analyses. Area under the curve (AUC) of the nasal shedding was determined using AUC function from Prism v6 (GraphPad Software, San Diego, CA, USA). AUC of each animal was calculated until 3 dpi and 6 dpi respectively; afterwards, mean and SD were calculated for each group.

### 2.7. Assessment of IFNγ Producing Cells

In order to evaluate secretion of IFNγ under different stimulations, an IFNγ Enzyme-Linked ImmunoSpot Assay (ELISPOT) was performed. Peripheral blood mononuclear cells PBMCs were isolated from 10 mL of EDTA-treated blood from all animals at different timepoints (0, 21, 39 days; 3 and 7 dpi). Cell isolation was done by a density gradient centrifugation using Histopaque 1077 (Sigma-Aldrich, Madrid, Spain), followed by an osmotic shock to remove the red blood cells. PBMCs were adjusted to 5 × 10^5^ cells/well and plated in cell culture plates with complete Roswell Park Memorial Institute (RPMI) 1640 medium (Lonza, Basel, Switzerland) supplemented with 10% FBS, 1% Glutamine, 1% penicillin-streptomycin and β-mercaptoethanol (Sigma-Aldrich, Madrid, Spain). Cells were incubated for 18 h at 37 °C 5% CO_2_ in precoated high binding 96-well plates (Costar Corning Incorporated, New York, NY, USA) with porcine IFNγ antibody (BD Pharmingen™, San José, CA, USA) in the presence of the following stimulus: Phytohemagglutinin (Sigma-Aldrich, Madrid, Spain) as positive control, recombinant Hemagglutinin 1 from A/Human/California/001/2009 strain (Sino Biologicals, Eschborn, Germany), UV inactivated virus A/Human/Catalonia/063/2009 and NG34 peptide. After incubation, plates were then washed to remove attached cells and stained with biotylinated IFNγ antibody (BD Pharmingen™, San José, CA, USA) and streptavidin (Invitrogen Life technologies. Madrid, Spain), followed by a development with insoluble TMB (Merck Life Science, Madrid, Spain). Resulting spots were counted under the Stereoscopic Zoom Microscope SMZ800 (Nikon Instruments Inc., Chiyoda, Japan). 

### 2.8. Humoral Immune Response Evaluation 

Humoral response was analyzed through an *in house* ELISAs against HA1 from A/Human/California/001/2009 and NG34 peptide in sera and BALF for total IgG, IgG1, IgG2 and IgA. High binding 96 well plates (Costar Corning Incorporated, New York, NY, USA) were coated with the analyte of interest in carbonate buffer and incubated overnight at 4 °C. After blocking the plates, samples were incubated 1 h at 37 °C and later stained with rabbit anti-pig IgG H + L HRP conjugated (Sigma-Aldrich, Madrid, Spain) to detect total IgG. With BALF samples, same antibodies including goat anti-pig IgA HRP conjugated (AbDSerotec, Oxford, UK) for IgA were included. IgG isotypes in sera were assessed staining the samples with mouse anti-pig IgG1 or mouse anti-pig IgG2 antibodies (Bio-Rad Laboratories, Madrid, Spain) followed by a staining step with goat anti-mouse IgG HRP conjugated (Sigma-Aldrich, MO, USA). Staining steps with antibodies were carried out for 1 h at 37 °C; after washes, plates were developed with soluble TMB (Sigma-Aldrich, MO, USA) and stopped with 1N H_2_SO_4_. Plates were read in a Power Wave XS spectrophotometer (Biotek Instruments, Winooski, VT, USA) at 450 nm wavelength. Swine IV HA1-positive and negative sera (GD Animal Health, Deventer, The Netherlands) and NG34-positive serum were included as internal controls for the technique. Thresholds of positive values were considered above the mean of the negative animals plus three times their standard deviation. 

### 2.9. Hemagglutination Inhibition and Neutralization Assays 

To assess the level of protecting antibodies, two different assays were performed: hemagglutination inhibition (HI) assay and neutralization (NT) assay in BALF with MDCK cells. For both procedures, protocols from WHO [22] and OIE [23] were followed and are briefly described below. Challenge strain A/Catalonia/63/2009 pdmH1N1 was used for both techniques as well as reference sera from GD Animal Health (Deventer, The Netherlands) were included as positive and negative controls.

A total of 5 mL of fresh blood was obtained from 3 chicken by cardiac puncture and mixed with Alsever’s solution (1:1). Red blood cells were washed twice with PBS centrifuging at 1115 rpm (250 g) for 10 min and adjusted with PBS to a final concentration of 50% for hemadsorption and 0.5% for hemagglutination and inhibition assays. 

Sera from all sampling timepoints were treated with RDE II Seiken (Denka Chemicals, Tokyo, Japan) for 18 h at 37 °C, followed by a heat-inactivation for 1 h at 56 °C and subsequent hemadsorption. Sera were two-fold diluted in PBS in a v-bottomed 96 well plate; 25 µL of viral antigen diluted to 4 Hemagglutination Units (HAU) was dispensed to each well and incubated for 1 h at room temperature. After that, 25 µL of 0.5% of red blood cells were added to the mixture; after 1 h, plates were tilted to evaluate hemagglutination. Antibody titers were considered as the reciprocal dilution where the inhibition was complete; seroprotective titers were considered above 1/40. 

For BALF neutralization (NT) assay, samples were heat inactivated at 56 °C for one hour and two-fold diluted in DMEM 1%Gln 1% P-ST and mixed with challenge virus with a TCID_50_/well of 100 for two hours at 37 °C at 5% CO_2_. After incubation, a post-infection medium (DMEM 1%Gln 1% P-ST) with the help of 10 µg/mL porcine trypsin (Sigma-Aldrich, Madrid, Spain) was added to plates and incubated for one week until examination for cytopathic effect. Titer was expressed as the reciprocal dilution where no cytopathic effect appeared.

### 2.10. Statistical Analysis

Graphs and statistical analysis were performed using Prism v6 (GraphPad Software, San Diego, CA, USA). Raw data was ln(log) transformed to reach gaussian distribution and confirmed using the Shapiro–Wilk test. Statistical differences were analyzed by ANOVA. Afterwards, post-hoc multiple comparisons between vaccinated groups and NV/C group were performed using Dunnett’s test. Statistical significance was denoted as it follows in each graph: * *p* < 0.05, ** *p* < 0.01, *** *p* < 0.001, **** *p* < 0.0001. 

## 3. Results

### 3.1. Clinical Signs and Pathology

**Figure 1:**
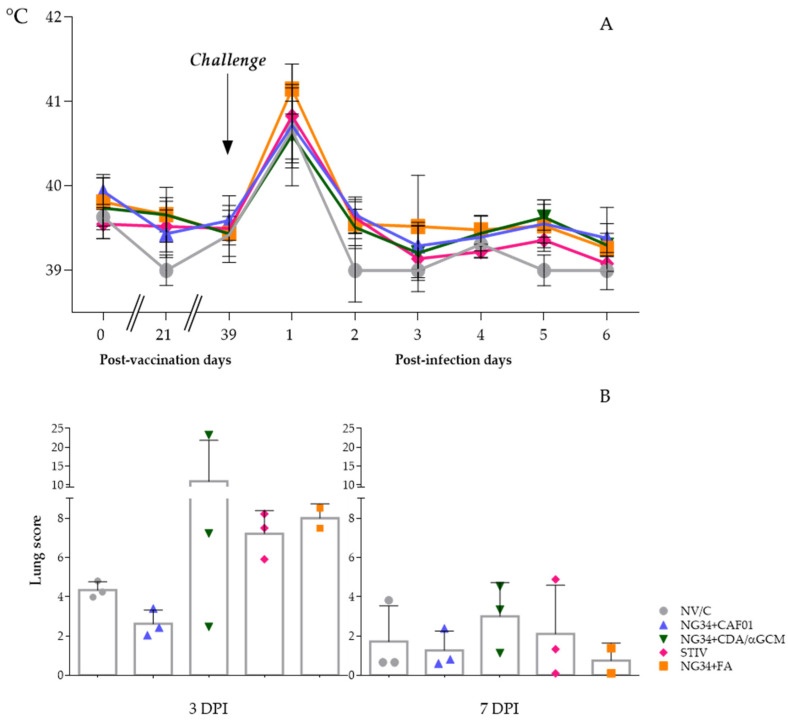
Graphs depicting average and SD of rectal temperatures taken during the experiment (**A**) and Influenza like pulmonary cranio-ventral consolidation lesions observed in animals euthanized at 3 and 7 days after the infection (**B**).

After the inoculation with pdm09 IAV, pigs did not display evident respiratory clinical signs. However, they developed a peak of pyrexia (rectal temperature >40 °C) and lethargy one day after the infection, without significant differences between groups (Figure 1A). This pyrexia was resolved two dpi and temperatures remained constant during the following days, where rectal temperatures remained below 40 °C until the termination of the study in all the animals. Dyspnea, coughing, abnormal breathing, nasal/ocular discharge or conjunctivitis were not observed during all the experimental procedure. No clinical signs or fever was observed in non-infected control pigs. 

At necropsy days, gross lesions characterized by pulmonary cranio-ventral multifocal consolidation were observed in lungs of inoculated animals. Numerically higher scores of typical flu-like areas were observed in animals euthanized at three dpi compared to animals sacrificed at seven dpi (Figure 1B). Moreover, broncho-interstitial pneumonia compatible with IV infection was confirmed in all inoculated animals at microscopic level but with no significant differences between them. Compared to the non-vaccinated group, pulmonary scores of the group vaccinated with NG34 + CDA/αGCM were highly variable on three dpi, having one animal with extremely severe lesions and another one where lung lesions were almost negligible. Although reduced by seven dpi, variability within the animals vaccinated with NG34 + CDA/αGCM remained higher in comparison to other vaccinated and challenged animal groups. On the contrary, IAV associated lung lesions in animals vaccinated with NG34 + CAF^®^01 were relatively homogeneous and considerably less severe in all animals both on three and seven dpi compared to pigs from the non-vaccinated group (Figure 1B). Animals vaccinated with TIV showed a high lesion score on three dpi that were greatly reduced on seven dpi. Difference between both timepoints was detected (*p* < 0.001). However, all the described variability and differences between groups were not statistically significant in both timepoints (*p* > 0.1). Tables with pictures of ventral and dorsal sides of lungs from infected animals are available in Supplementary Data (Tables S1–S4).

### 3.2. RT-qPCR Results–Viral Load

**Figure 2:**
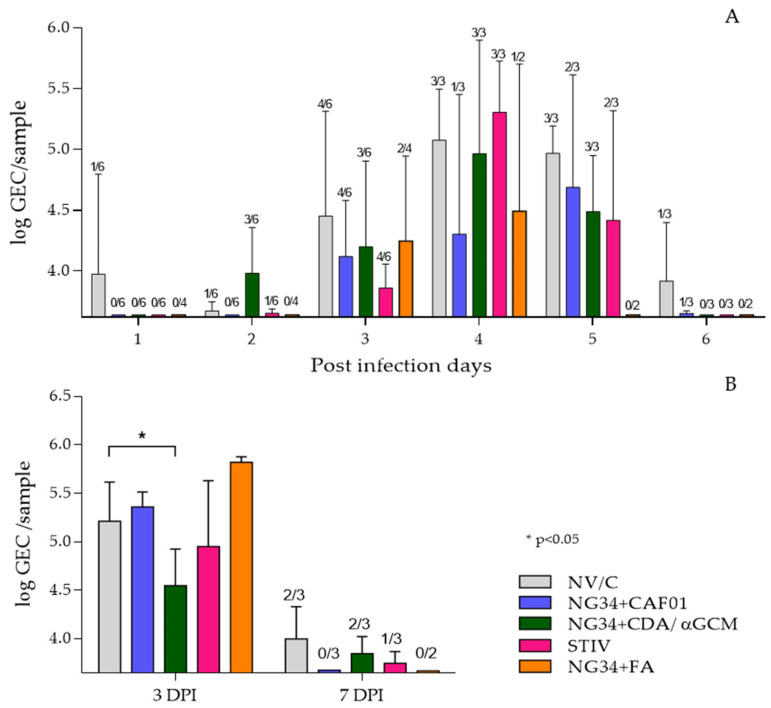
Results obtained by RTqPCR analysis from nasal swabs (**A**) and bronchoalveolar lavage (**B**). Viral load in nasal swabs is represented as log GEC/sample of Influenza M protein gene from purified RNA of nasal swabs or BALF. Bars express averages and error bars the SD of each group. The number of animals with positive signal in qRT-PCR are represented above each bar in the figure.

RT-qPCR was used to explore nasal viral shedding and viral load in BALF from studied animals. All groups exhibited similar pattern of nasal virus load having a peak 4 dpi with virus titers in relative numbers around Log_10_ 5 GEC (genome equivalent copies). Nonetheless, 2 out of 3 animals of the group vaccinated with NG34 + CAF^®^01 had undetectable viral genome levels at four dpi. This animal group also showed relatively lower virus secretion during the whole experimental infection period, except for one animal that presented high viral load in nasal swabs on day 4 and 5 post-challenge (Figure 2A). The other groups, vaccinated with seasonal TIV or NG34 in combination with CDA/αGCM or Freund’s adjuvants, showed a decreasing trend in nasal viral load from day five onwards compared to control non-vaccinated challenged animals. None of these results were statistically significant (*p* > 0.1) (Figure 2A). 

Regarding AUC calculation, all vaccinated groups had a lower value than the NV/C group at 3 dpi, being the TIV group the one with the lowest AUC. Same effect was observed at 6 dpi, when the NG34 + FA group presented the lowest AUC. However, these results were not significantly different (*p* > 0.1) among groups (Table 2). 

Virus load in the BALF from animals sacrificed on 3 dpi was quite homogenous in all vaccinated infected animals and no significant differences between vaccinated or control non-vaccinated infected animal groups was detected, with the exception of animals receiving NG34 + CDA/αGCM that showed significantly reduced (*p* < 0.05) viral loads (Figure 2B). A noteworthy difference was observed on day 7 p.i. where the virus load was below detection levels in all pigs from the NG34 + CAF^®^01 vaccinated group (two-way ANOVA *p* < 0.0001; Dunnett’s test *p* < 0.05). These results were further confirmed by analyzing virus load in the lung tissues homogenates taken on days 3 and 7 post-challenge. Where a residual presence of viral genome was detected albeit GEC were extremely low at day 7 after infection in all animal groups (data not shown).

### 3.3. Cell Immune Response-ELISPOT

Vaccine induced IV specific-T cell response was evaluated by measuring IFNγ producing cells by ELISPOT. PBMCs were harvested at defined time points before and after challenge from non-vaccinated and vaccinated (NG34 + CAF^®^01, NG34 + CDA/αGCM, NG34 + FA and TIV) animals and were subjected to different stimulus (UV inactivated pdm09 H1N1, HA1 or NG34) in vitro. The number of IFNγ producing cells varied depending on the stimulus used. 

**Figure 3:**
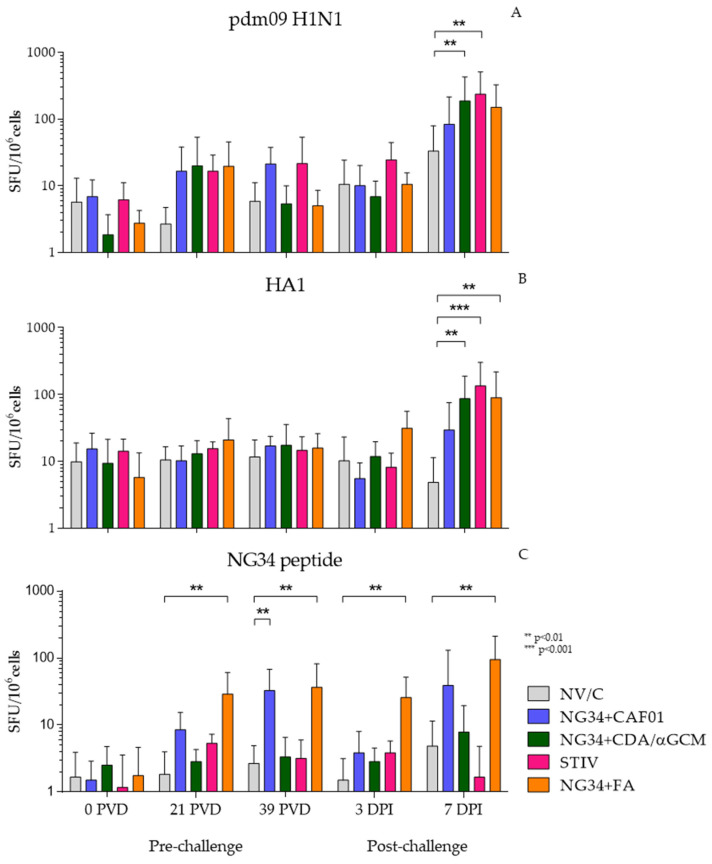
IFNγ ELISPOT results obtained in stimulated PBMCs from blood collected during the study with UV inactivated pdm09 H1N1 A/Human/Catalonia/063/2009 (**A**); recombinant HA1 (**B**) and NG34 peptide (**C**).

PBMCs isolated from all vaccinated animal groups showed an increase in the number of IFNγ producing cells stimulated in vitro with inactivated pdm09 H1N1 virus (Figure 3A), HA1 (Figure 3B) and NG34 (Figure 3C) on day seven post-challenge. These increments were statistically significant only in NG34 + CDA/αGCM and STIV groups (*p* < 0.0001) compared to non-vaccinated, challenge control animal group. Similarly, PBMCs stimulated in vitro with purified HA1 protein from pdm09 H1N1 only reacted against the antigen 7 days after the infection with significant differences with respect the non-vaccinated animals in NG34 + CDA/αGCM, NG34 + FA and TIV groups. 

Under NG34 peptide stimulation, the animals vaccinated with NG34 + FA reacted progressively throughout the different vaccination and after challenge timepoints and becoming significant again (*p* < 0.01), as the rest of the stimulus on day 7 after infection. Moreover, only groups vaccinated with NG34 + CAF^®^01 or Freund’s adjuvant reacted against the antigen and with notable difference in comparison with the first timepoint, although for NG34 + CAF^®^01 the differences were only statistically significant before the challenge (39 PVD). 

### 3.4. Antibody Response 

**Figure 4:**
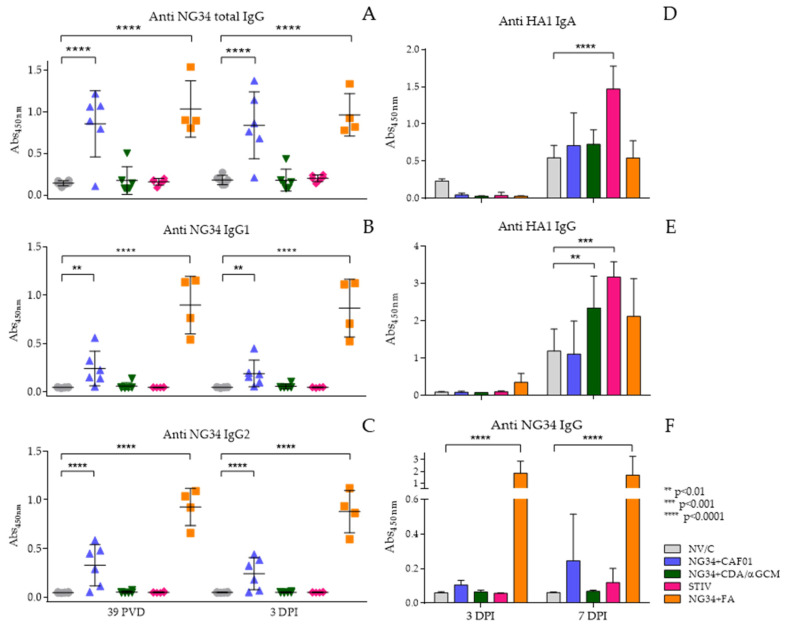
Antibody response in sera and BALF. Total Anti NG34 IgG (**A**), Anti Ng34 IgG1 (**B**), Anti NG34 IgG2 (**C**), Anti HA1 IgG in BALF (**D**), Anti HA1 IgA in BALF (**E**) and Anti NG34 IgG (**F**).

Antibody response was analyzed against the NG34 epitope and the complete HA from the pdm09 H1N1 IAV (Figure 4). A significantly increased NG34-specific IgG response, however, was noted in animal groups vaccinated with NG34 + CAF^®^01 similar to NG34 + FA. This response remained elevated after challenge and displaying statistically significant differences (*p*  <  0.0001) compared to the non-vaccinated challenged group (Figure 4A). The NG34 + CDA/αGCM vaccinated group showed a weak IgG response against NG34 with only one animal responding higher than background levels. None of the remaining vaccinated groups showed NG34-specific IgG response. This trend remained similar after the challenge where significantly higher IgG response against NG34 antigen was only observed in animals vaccinated with NG34 + CAF^®^01 and NG34 + FA (Figure 4A). Further analysis of antibody isotype revealed that both the NG34-specific IgG1 and IgG2 were significantly elevated in pigs from the animal group vaccinated with NG34 + CAF^®^01 and NG34 + FA (Figure 4B,C). The rest of the vaccinated groups (NG34 + CDA/αGCM, STIV) were barely inducing IgG1 or IgG2 titers. Systemic HA1-specific IgG titers were only observed in animal group vaccinated with TIV after challenge with pdm09 H1N1 (data not shown).

Presence of antibodies in BALF at necropsy time points was also evaluated. HA1-specific IgA and IgG in BALF in all animal groups were observed only 7 days after challenge (Figure 4D,E). HA1-specific IgG and IgA response in BALF was significantly higher in the group vaccinated with TIV (*p* < 0.001) than the rest of vaccinated animals. Vaccination with NG34 + CDA/αGCM also induced a statistically significant HA1-specific IgG response in BALF (*p* < 0.01), but no IgA response could be detected in the BALF collected from animals vaccinated with NG34 + CDA/αGCM (Figure 4E). None of the vaccinated groups except NG34 + FA showed specific NG34-IgG response on day 3 after challenge in the BALF. A positive signal, however, could be detected on day seven after challenge in the BALF collected from animals vaccinated with NG34 + CAF^®^01 (Figure 4F). 

### 3.5. Hemagglutination Inhibition and Neutralizing Antibodies

HI and NT antibody titers were evaluated in the sera and BALF collected from vaccinated and non-vaccinated animal groups 7 days after the challenge with pdm09 H1N1. NT titers were either negative or low in non-vaccinated challenged animal group. All other groups vaccinated with NG34 + CAF^®^01, NG34 + CDA/αGCM, STIV or NG34 + FA showed a positive reaction in the NT assay albeit with lower intensity and variations within and among the groups. However, STIV vaccinated animals showed higher NT titers in comparison with the rest of experimental groups (Table 3). 

HI antibody titers, on the other hand, were increased a bit in the non-vaccinated challenged control animal up to 80 due to the effect of pdm09 H1N1 infection. HI titers in the rest of experimental groups were in general increased (2–3 fold) but outstandingly in STIV vaccinated animals (14-fold in proportion comparing with NV/C group) (Table 3).

## 4. Discussion

Adjuvant formulations are necessary components of modern vaccines based on subunit proteins/peptides, which are often poorly immunogenic without additional immune stimulants [24]. On the other hand, different antigen structures may be affected by adjuvant formulations such as emulsions. For instance, virus-like particle antigens may interact with adjuvant formulations in very different ways compared with recombinant subunit proteins or immunogenic peptides [25]. The use of adjuvants can also result in a skewing of the resulting humoral or cellular immune response [26,27]. This can in turn improve or reduce vaccine efficacy or even promote immune pathological reactions (e.g., antibody dependent enhancement (ADE), vaccine-associated enhanced respiratory disease (VAERD) [28,29]). 

In this study, we assessed the immune responses to pdm09 H1N1 IV infection in pigs vaccinated with HA1 peptide NG34 adjuvanted with CAF^®^01 or CDA/αGCM. The NG34-specific IgG response was significantly elevated in the sera collected from pigs vaccinated with NG34 + CAF^®^01 and was observed before and after challenge with pdm09 H1N1 IAV. Both the IgG1 and IgG2 isotypes were produced after vaccination with NG34 + CAF^®^01 being IgG2 isotypes more dominant than IgG1, in contrast to infection with live virus that generated a more balanced and broader immune response. Strong IgG2 response was observed also in a previous study in mice immunized with NG34 + CAF^®^01 [12] and it is in line with other recently published studies using combination of different antigens with CAF^®^01 [13,30]. Previously, it has been suggested that IgG2 response is vital in protection against IV, particularly in the absence or low amount of virus neutralizing antibodies [31,32]. Pigs vaccinated with the NG34 + CAF^®^01 combination also generated relatively good HI titers although variations within the animal group was observed. Similarly, the NG34 + CAF^®^01 vaccinated group also showed NT titers in BALF, albeit with variations and at low levels. More importantly, NG34 + CAF^®^01 vaccinated group showed relatively lesser pulmonary lesion scores and reduced virus load in the BALF as well as lower virus shedding after pdm09 H1N1 infection. These results are in line with the ones obtained in ferrets with TIV combined with CAF^®^01, where protection against heterologous IAV was observed in an HIA-independent manner [15]. NG34-specific IgG titers in pigs vaccinated with NG34 + CDA/αGCM were relatively low. Moreover, no NG34-specific IgG1/IgG2 isotype response was observed in pigs vaccinated with this latter combination, except one animal that showed relatively higher IgG titers in sera collected both at pre- and post-challenge time points in this group. However, this group was the only one showing a statistically significant reduction of viral load in BALF on day three pi. This can be explained, at least in part, by the fact that NG34 + CDA/αGCM and STIV were the only groups in which it was observed a significant increment in HA1-specific antibodies in BALF, as well as H1N1 and HA1 specific IFNγ-producing cells. In terms of lung pathology, this group immunized with NG34 + CDA/αGCM combination had a very variable pathological score, even higher in some individuals than the non-vaccinated challenged group. This was also true, to a lesser extent, for the animals in the STIV and NG34 + FA groups. Interestingly, similar outcomes, including an increased viral shedding were reported in a pig experiment with animals immunized intranasally with αGCM before a challenge with pandemic H1N1 A/California/04/2009 [33]. On the other hand, CDA-adjuvanted vaccines against IV have provided efficient protection [34]. The so-called VAERD effect is defined as an undesirable side effect described in pigs with some inactivated-based Influenza vaccines, characterized by an exacerbation of the severity of the IV induced disease, including long lasting fever, clinical signs and an increase of lung consolidated areas [35]. Despite the fact that we cannot relate these extended pulmonary lesions to a VAERD effect due to the low number of animals used in this study, we considered that this is an issue to further study in regards the αGCM/antigen combination. 

In response to in vitro stimulation of PBMC with inactivated pdm09 H1N1 influenza virus, we detected a peak of IFNγ-producing cells in all vaccinated pigs, including with the STIV vaccine, 7 days after challenge with the pdm09 H1N1 virus. The lack of IFNγ response observed in pigs vaccinated with the non-adjuvanted TIV vaccine (even following the booster vaccination) reflects the incapacity or at least the low efficiency of the non-adjuvanted vaccines to elicit an influenza-specific lymphocyte T response in these pigs as it was reported previously in ferrets [14]. Such an anamnestic response in the number of influenza-specific IFNγ-producing cells in the blood has similarly been detected only at day 7 after the challenge of pigs with A/Sw/Indiana/1726/88 H1N1 swine influenza virus [36]. It has been previously shown that some adjuvants have the ability to strongly enhance antigen cross-presentation (including that of peptide or protein antigen) [37,38,39]. CAF^®^01 adjuvant contained in the adjuvanted NG34 vaccine induce local inflammation and recruitment of various innate immune cells as has been reported for other adjuvant ASO3 [40], although their mechanism of action is different in terms of depot formation [41]. This depot formation produced in the tissue by CAF^®^01 induces a strong cell response involving CD4+ T cells [42] and may similarly enhance antigen cross-presentation with the subsequent CD8+ cytotoxic T cell proliferation. Only NG34 combined with CAF^®^01 vaccine elicited systemic humoral responses as well as an enhanced cell-mediated immune response 7 days after pdm09 H1N1 challenge. However, pigs vaccinated with this combination, although having reduced viral load and cleared the virus earlier, were unable to significantly limit nasal virus secretion, as viral RNA continued to be detected in the nasal cavity. In this respect, nasal shedding progressed as expected in a similar way as it occurs in untreated animals [43].

It has previously been shown that pigs with an HI antibody titer equal to or above 20 were generally protected from a subsequent influenza challenge [44]. Even though all vaccinated animal groups (CAF^®^01, CDA/αGCM, FA, STIV) exhibited HI titers above 40, they were not significantly protected. It is still unclear how the systemic responses generated after vaccination correlate with local mucosal responses in the respiratory tract that may also contribute to reduction in virus shedding [45]. Interestingly, a similar study conducted in ferrets challenged six weeks after the initial vaccination, generated results consistent with our own findings with NG34 + CAF^®^01 vaccine. This study showed that pdm09 H1N1 vaccines reduced (adjuvanted split vaccine) or had no effect (non-adjuvanted whole vaccine) on the viral shedding from the upper respiratory tract, although the adjuvanted split vaccine did prevent viral replication in the lower respiratory tract of ferrets [46,47]. This suggest that local immunity may play a role for viral shedding but less for pulmonary disease. This could, e.g., be obtained by priming parenterally to obtain the systemic immunity important for prevention of pulmonary disease and boost intranasally to obtain local immunity in the nasal cavity and thus avoid viral shedding. This prime-pull strategy has previously been described for CAF^®^01 in mice [48] and pigs [49].

Immunization with NG34 + CDA/αGCM under the current experimental conditions, on the contrary, appeared less effective than immunization with NG34 + CAF^®^01 both in inducing adequate immune response and limiting pathological outcome after pdm09 H1N1 challenge. Specific NG34 anti-IgG were only observed in one animal of the group immunized with NG34 + CDA/αGCM before and after the IAV challenge. Similarly, increased number of viral particles in nasal swabs during the experiment and in BALF collected on day seven after challenge as well as extended lung consolidated lesions on day three after the infection with pdm09 H1N1 IV were observed in this group. These findings contrast to the ones from a study published by Khatri et al. [50], where protective effect of αGalCer adjuvant, a component also included in the adjuvant used in our study, against Swine Influenza Virus (SwIV) infection in pigs has been demonstrated. In this study, however, the authors used a UV-inactivated SwIV together with αGalCer as vaccine. Thus, the CD1d agonist αGCM in combination with CDA might be a suboptimal adjuvant for a short peptide-based vaccine. Moreover, the route of application (intranasal), age and type of piglets and particularly the concentration of αGalCer used in this study was also different to the one presented here. The authors could document that protection against SwIV infection in pigs vaccinated with UV-inactivated SwIV + αGalCer correlated with the αGalCer concentration used. Likewise, another study from Artiaga et al. [51], reported that αGalCer protects pigs from IV infection when administered as vaccine adjuvant and attributed the observed protection to enhanced NKT-cell concentrations resulted after administration of vaccine containing αGalCer. A more recent study report by Gu et al. [33], in contrast, suggested that increased NKT-cells does not alter disease outcome in pigs prophylactically treated with αGalCer. In all these reports, unlike our study, αGalCer was either used alone or as an adjuvant with antigen combination. At this stage, we cannot rule out that the combination of CDA/αGCM used in our study with NG34 might have an antagonistic effect on activation of immune cells, as also suggested in a study published by Matos et al. [52] demonstrating that protection against *Trypanosoma cruzi* infection in mice is more efficient when only CDA rather than αGCM is used as adjuvant together with Tc52 antigen. In all cases, this hypothesis needs further research to be ascertained. For this reason, we consider that optimization of dose in the combination of CDA and αGCM adjuvants would be needed in order to better determine its efficacy and immunogenicity. 

In summary, this study helped to report the outcomes in pathology, cell-mediated and humoral immune responses resulting from the vaccination with the novel vaccine formulations and the subsequent infection. Unfortunately, NG34-combinations formulated for this study did not afford the immune correlates of protection achieved by the STIV, nowadays accepted for the licensed vaccines. However, we admit that the number of animals per group used in our study was rather small to make statistically relevant conclusions. A higher number of parameters like route of application, age of animals, concentration of adjuvants, antigen choice, different challenge strains and in particular, sufficient number of animals should be considered in future experiments. These issues will help in the future to obtain conclusive and statistically significant results to dissect the relevant parameters for the induction of protective immune responses against IV infection in pigs.

## 5. Conclusions

Pigs vaccinated with NG34 adjuvanted with CAF^®^01 or CDA/αGCM reacted differently upon IAV infection regarding pathological outcome, viral shedding and cell-mediated and humoral responses. Thus, pigs immunized with NG34 in combination with CAF^®^01 seroconverted against the antigen, had numerically lower lung lesion score, decreased viral shedding and increased of IFNγ producing cells. However, further studies scaling-up the number of animals would be needed to confirm the effectivity and immunogenicity of these combinations of adjuvants and antigens against IAV infection. 

## 6. Patents

C.A.G. and T.E. are named as inventors in a patent covering the use of CDA as a neonatal adjuvant (EP 19193982), which was previously patented covering the use of CDA as adjuvant (PCT/EP 2006010693). Moreover, C.A.G. and T.E. are named as inventors in a patent covering the use of αGCM as adjuvant (EP 2005022771.9).

## Figures and Tables

**Table 1:** Distribution of experimental groups and composition of the different antigen and adjuvant combinations.

Experimental Group	N	Antigen	Adjuvant
1. Unvaccinated/unchallenged (NV/NC)	6	PBS	None
2. Unvaccinated/challenged (NV/C)	6	PBS	None
3. NG34–CAF^®^01	6	50 µg of NG34	980 µL of CAF^®^01
4. NG34–CDA/αGalCerMPEG (CDA/αGCM)	6	50 µg of NG34 *	25 µg of CDA + 25 µg αGCM
5. NG34–Freund’s Adjuvant (FA)	4	50 µg of NG34 *	600 µL of CFA/IFA ^1^
6. Seasonal Trivalent Influenza Vaccine (STIV)	6	500 µL of *Chiroflu^®^* 2018–19 seasonal vaccine ^2^	

One mL of each vaccine formulation per animal was injected except for STIV, where the dose consisted of 0.5 mL. *: Vaccine antigen diluted in PBS. ^1^: First immunization was performed using Complete Freund’s Adjuvant (CFA); boost was prepared using Incomplete Freund’s Adjuvant (IFA). ^2^: Including hemagglutinins from: A/Singapore/GP1908/2015 (similar strain to A/Michigan/45/2015 (H1N1) pdm09); A/Singapore/INFIMH-16-0019/2016 (H3N2) and B/Maryland/15/2016 wild type (similar strain to B/Colorado/06/2017).

**Table 2:** Area under the curve calculation of the nasal shedding performed until 3 dpi and 6 dpi.

Experimental Group	AUC Until 3 dpi (*n* = 6)		AUC Until 6 dpi (*n* = 3)	
	Mean	SD	Mean	SD
Unvaccinated/Challenged (NV/C)	0.6052	±0.6680	4.1520	±0.9719
NG34 + CAF^®^01	0.2397	±0.2316	2.2317	±2.2538
NG34 + CDA/αGCM	0.6228	±0.6968	2.8888	±2.3776
Seasonal Trivalent Influenza Vaccine (TIV)	0.1255	±0.1180	2.5247	±0.4745
NG34 + FA *	0.3036	±0.3506	1.4695	±2.0782

* NG34 + FA group: AUC calculated until 3 dpi (*n* = 4), 6 dpi (*n* = 2).

**Table 3:** HI titers in sera and neutralization in BALF from individual animals euthanized at 7 dpi (3 animals in each group except for the NG34 + FA group, with 2 pigs).

Experimental Group	HI Antibody Titer in Sera *	Neutralization Antibody Titer in BALF *
Unvaccinated/Challenged (NV/C)	80	20
	80	40
	80	30
NG34 + CAF^®^01	320	0
	80	30
	160	120
NG34 + CDA/αGCM	160	80
	160	40
	80	20
Seasonal Trivalent Influenza Vaccine (STIV)	160	120
	640	30
	2560	100
NG34 + FA	160	30
	320	30

*: Titers expressed against challenge strain A/Catalonia/63/2009 pdmH1N1.

## Data Availability

Not applicable.

## Supplementary Materials

The following are available online at https://www.mdpi.com/article/10.3390/vaccines9070751/s1, Table S1. Ventral pictures of the lungs from challenged animals sacrificed at 3 dpi. Table S2. Dorsal pictures of the lungs from challenged animals sacrificed at 3 dpi. Table S3. Ventral pictures of the lungs from challenged animals sacrificed at 7 dpi. Table S4. Dorsal pictures of the lungs from challenged animals sacrificed at 7 dpi.
