# Supplementary material for: Immune Responses to Pandemic H1N1 Influenza Virus Infection in Pigs Vaccinated with a Conserved Hemagglutinin HA1 Peptide Adjuvanted with CAF®01 or CDA/αGalCerMPEG
Source: Vaccines (Basel). 2021 Jul 6;9(7):751. doi: 10.3390/vaccines9070751 (PMC8310093; doi:10.3390/vaccines9070751)
Supplement: Supplementary file 1 [file vaccines-09-00751-s001.zip › vaccines-1219568-supplementary.pdf]

Table S1. Ventral pictures of the lungs from challenged animals sacrificed at 3 dpi.

**Group**	**Animals**		
NV/C	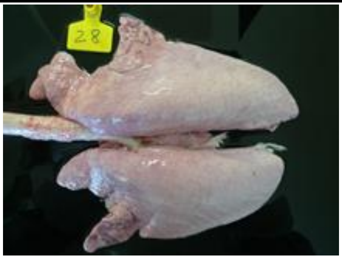	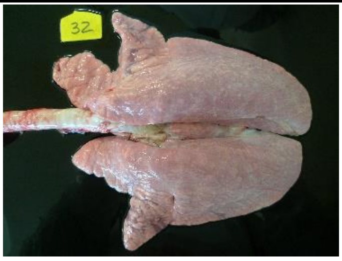	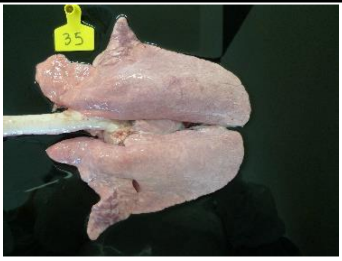
NG34+CAF®01	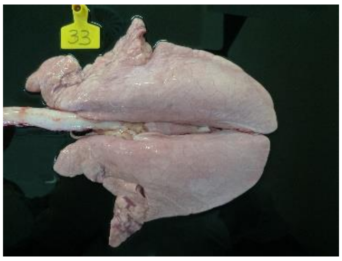	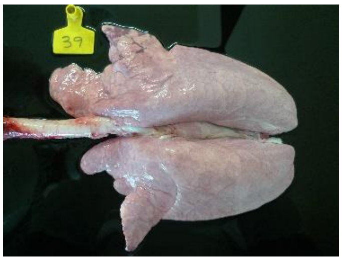	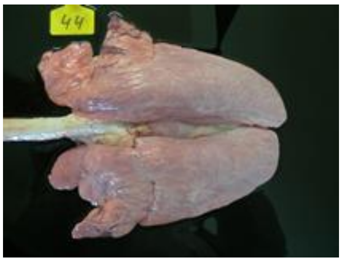
NG34+CDA/αGCM	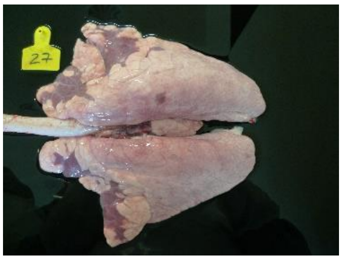	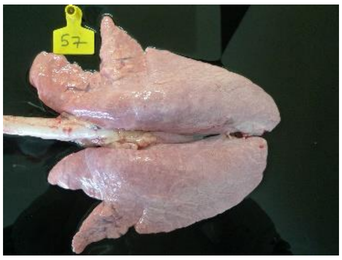	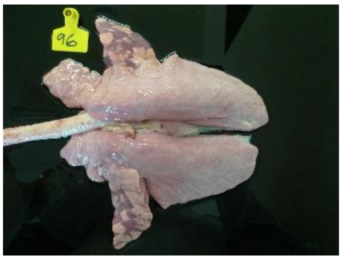
STIV	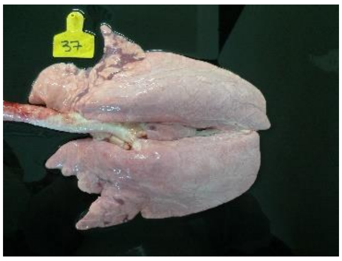	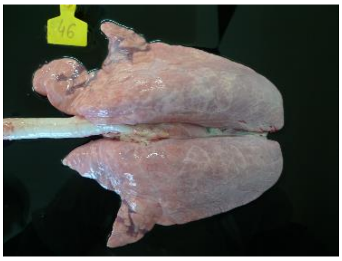	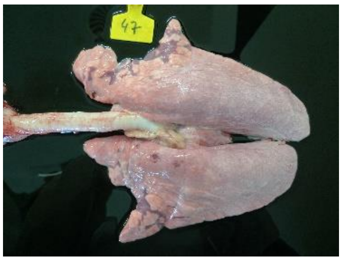
NG34+FA	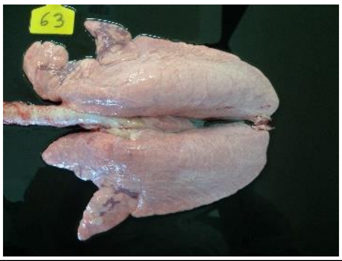	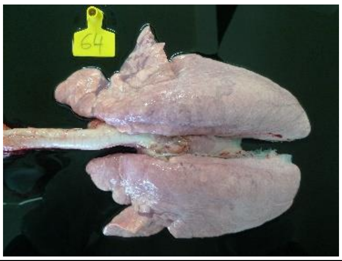	

Table S2. Dorsal pictures of the lungs from challenged animals sacrificed at 3 dp

Group		Animals	
NV/C	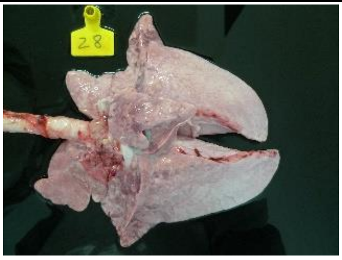	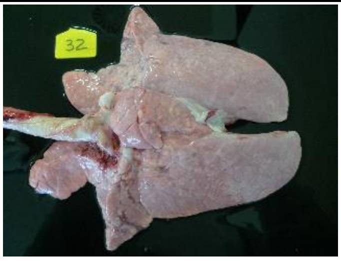	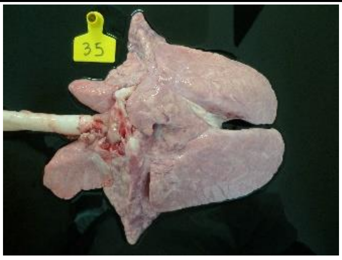
NG34+CAF®01	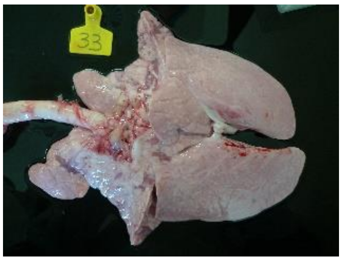	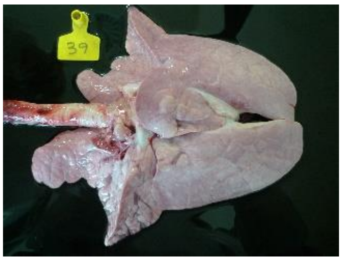	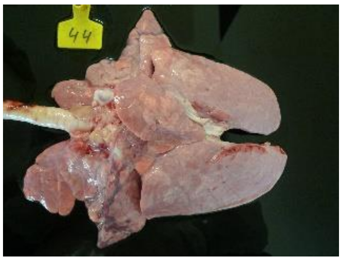
NG34+CDA/αGCM	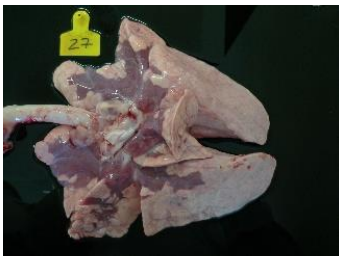	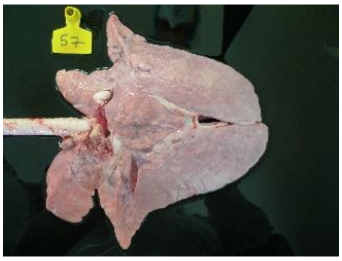	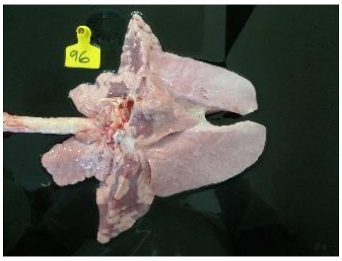
STIV	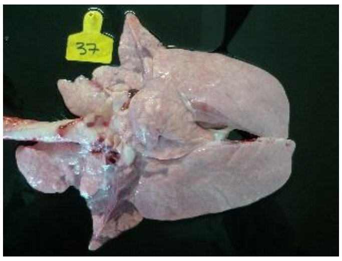	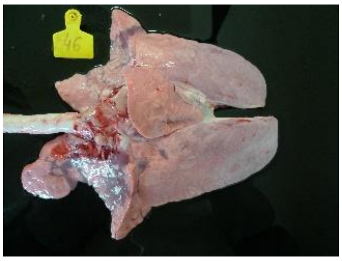	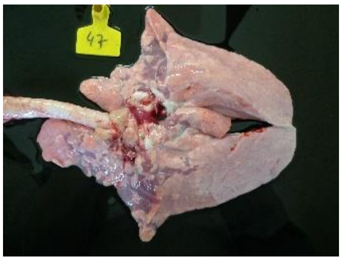
NG34+FA	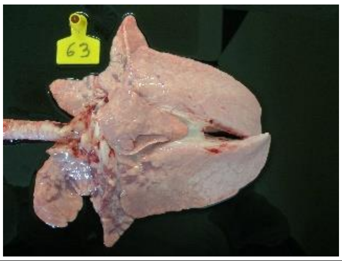	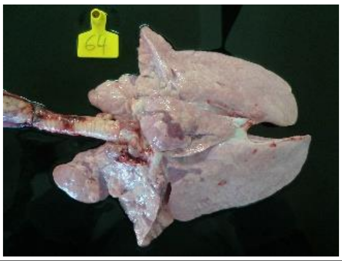	

Table S3. Ventral pictures of the lungs from challenged animals sacrificed at 7 dpi.

**Group**	**Animals**		
NV/C	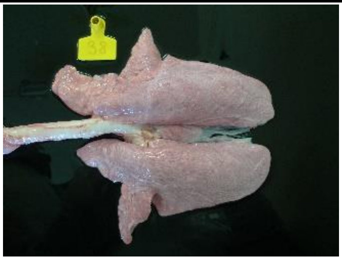	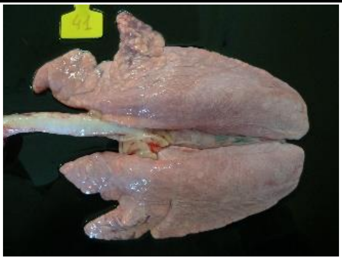	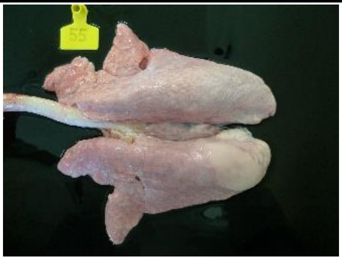
NG34+CAF®01	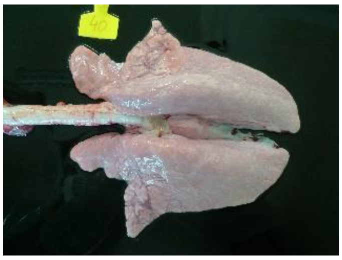	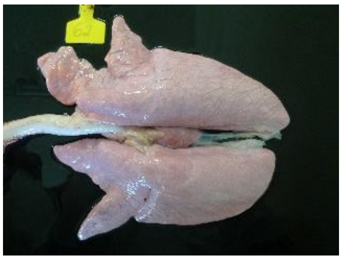	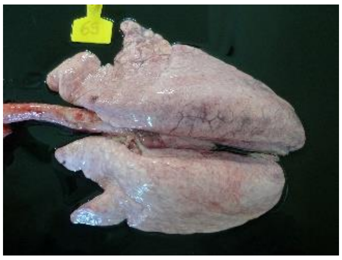
NG34+CDA/αGCM	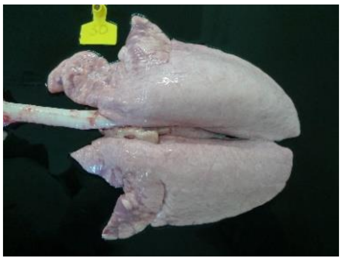	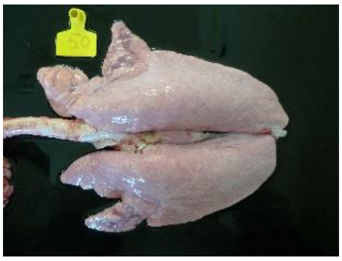	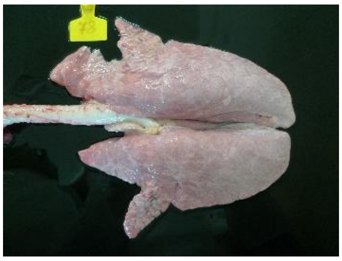
STIV	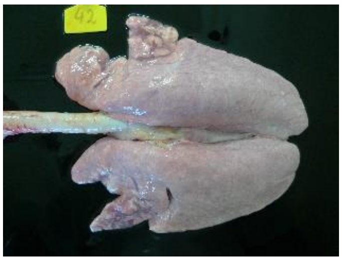	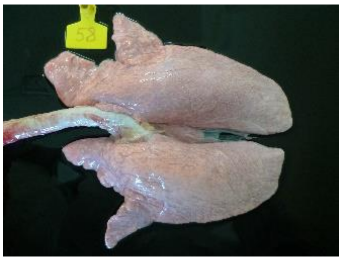	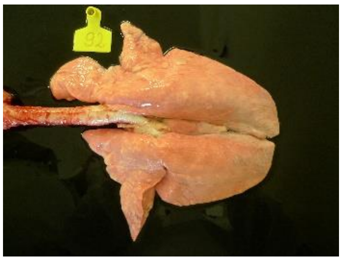
NG34+FA	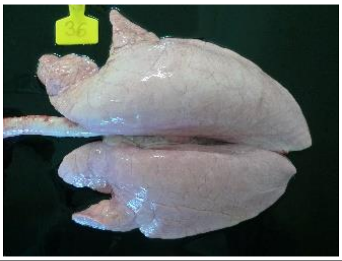	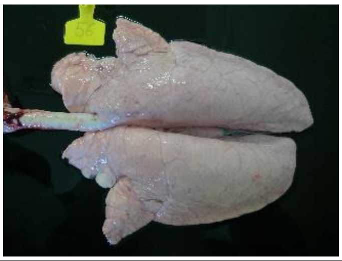	

Table S4. Dorsal pictures of the lungs from challenged animals sacrificed at 7 dpi.

**Group**		**Animals**	
NV/C	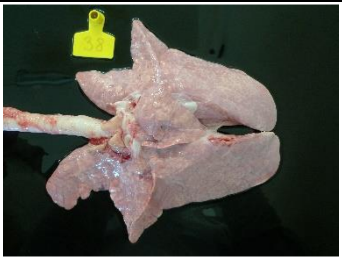	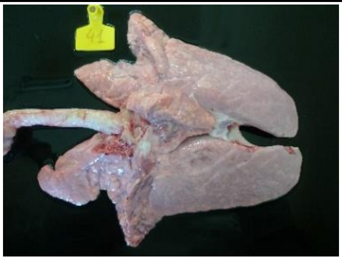	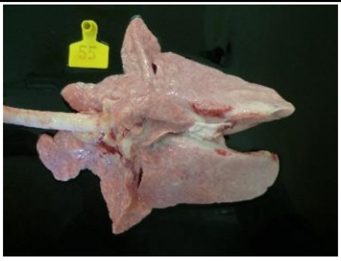
NG34+CAF®01	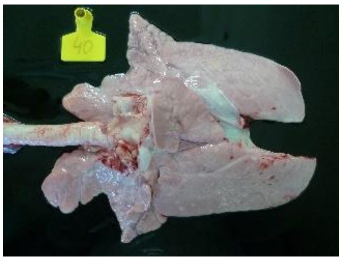	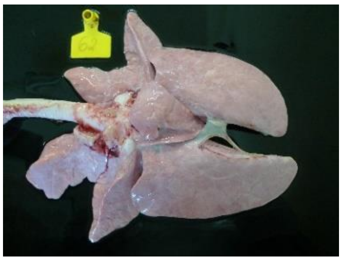	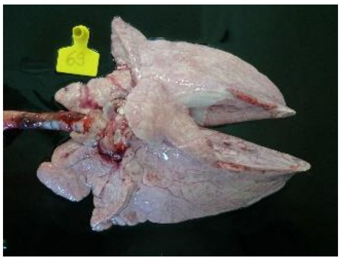
NG34+CDA/αGCM	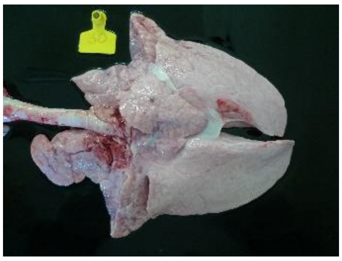	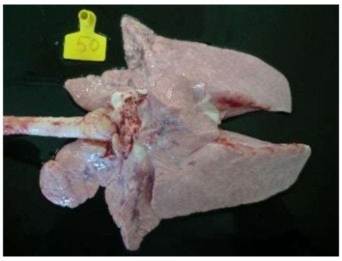	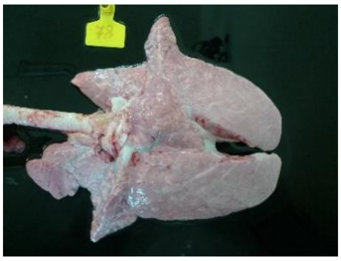
STIV	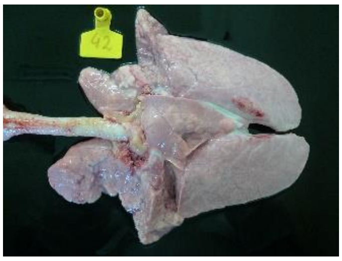	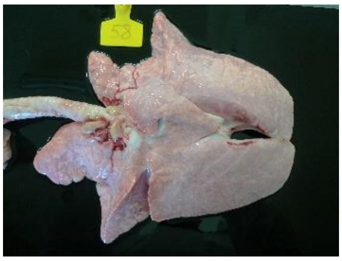	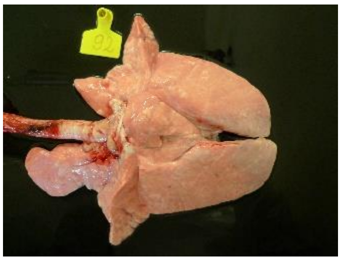
NG34+FA	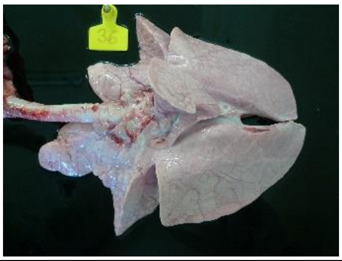	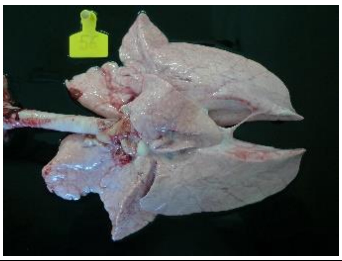	
